# Dietary apidaecin Api-PR19 addition enhances growth performance by regulating gut health and microbiota in broilers

**DOI:** 10.5713/ab.23.0357

**Published:** 2024-04-01

**Authors:** Chenxu Wang, Xinrui Wang, Rui Liu, Jiyang Min, Xiaojun Yang, Lixin Zhang

**Affiliations:** 1 College of Animal Science and Technology, Northwest A&F University, Yangling, Shaanxi 712100, China; 2 College of Life Sciences, Northwest A&F University, Yangling, Shaanxi 712100, China

**Keywords:** Antibacterial Peptide, Broiler, Growth Performance, Gut Microbiota, Intestinal Health

## Abstract

**Objective:**

This study investigated the effects of Apidaecin Api-PR19 as feed additive on growth performance, intestinal health, and small intestinal microbiota of broilers.

**Methods:**

A total of 360 1-d-old Arbor Acres broilers were randomly assigned to 3 groups with 6 replicates including control group with basal diet (CON), antibiotic growth promotor group with basal plus 10 mg/kg colistin sulfate and 50 mg/kg roxarsone (AGP), and antibacterial peptide group with basal diet plus 330 mg/kg Apidaecin Api-PR19 (ABP). The trial lasted 35 d.

**Results:**

Results showed that dietary Api-PR19 addition increased (p<0.05) the average daily feed intake, average daily gain and decreased (p<0.05) feed conversion ratio (FCR) during 1 to 21 d compared with the CON group. The digestibility of dry matter and crude protein were higher in AGP and ABP groups (p<0.05) where greater trypsin activity was detected in duodenum (p<0.05). The ratio of villus height to crypt depth (V/C) in duodenum and jejunum was increased at 35 d when broilers were given diets with ABP or AGP (p<0.05). Besides, ABP treatments up-regulated (p<0.05) the mRNA expression of *EAAT3*, *GLUT2*, *ZO-1*, and *Claudin-1* in duodenum of broilers at 35 d of age. The results of immunohistochemistry showed that ABP treatment significantly increased (p<0.05) duodenal secretory immunoglobulin A (sIgA) content. In addition, 16S rRNA gene sequencing revealed that there were differences in the intestinal microbiota diversity and composition among three groups. Notably, the linear discriminant analysis effect size showed that *p_Firmicutes*, *g_Enterococcus*, *g_Carnobacterium*, *g_Kitasatospora*, and *g_Acidaminococcus* were dominant in ABP group. Redundancy analysis showed that these changes in gut microbiota in ABP group had correlation with growth performance, intestinal morphology, and content of sIgA.

**Conclusion:**

In general, these results indicated that dietary 330 mg/kg Apidaecin Api-PR19 supplementation promoted growth performance of broilers by improving intestinal development, nutrients absorption, immune function and modulating intestinal microbiota.

## INTRODUCTION

Host biology is contextual to the coexisting microorganisms, which are living in the digestive tract and affect various physiological functions [1]. Intestinal diseases caused by conditional pathogenic were common in chickens such as *Escherichia coli*, *Salmonella*, and *Clostridium perfringens* [2]. The spread of gastrointestinal pathogens not only lead to an increasing morbidity and financial loss in broiler farms but also harmed human health through the food chain [3]. In the past few decades, subtherapeutic dose antibiotics are often used as feed additives for prevention of infectious diseases and growth promoters. However, the overuse of antibiotic growth promoters (AGPs) in animal feeding generates negative influences such as the imbalance of intestinal flora, evolution and selection of antibiotic-resistant bacteria and drug residues [4]. Therefore, the AGPs in poultry feed have been gradually forbidden in many countries.

To meet the increasing global demand for alternatives to AGPs, many novel and green feed additives had been developed to maintain intestinal health and promote growth performance of broilers [5]. Antibacterial peptide (ABP) is a kind of minor polypeptide with antimicrobial activity which could be produced by bacteria, plants, amphibians, mammals and insects upon pathogen infection and plays an important role in the innate immune system. The main methods to produce ABPs include extraction and purification from organisms, chemical synthesis, and construction of genetically engineered bacteria. Many studies have shown that ABPs have natural inhibitory effects on bacteria, fungi, and viruses without developing antibiotic resistance, which makes it one of the ideal substitutes for AGPs [6].

Apidaecin used in the study is a series of proline-rich, 18 to 20 residue small peptides isolated from lymph fluid of honeybees infected with bacteria. Unlike most conventional amphipathic ABPs, Apidaecins are non-amphipathic and have better membrane penetration ability, because they could combine with the membrane of bacteria in a non-specific way, followed by invasion into the periplasmic space and passing through the inner membrane with a receptor/docking molecule. Finally, apidaecin is transported into the interior of the cell, where it performs its bacteriostatic function [7]. What’s more, apidaecins are not likely to be toxic to animal cells and develop little or no bacterial resistance, which provides a good basis for future economic production of recombinant apidaecin [8]. Due to the natural antimicrobial and low propensity for the development of bacteria resistance properties, ABPs have been increasingly selected to be the candidates to replace AGPs in poultry industry.

Some studies have shown that apidaecin has positive effects on growth performance and health in animals [9]. However, the effects of Api-PR19 on the small intestinal microbiota and the relationship among growth performance, intestinal health and intestinal microbiota shaped by Api-PR19 remained unclear. In this study, we evaluated the effects of recombinant Apidaecin Api-PR19 as alternative to AGPs on growth performance, nutrient digestibility and gut morphology, mRNA expression of nutrient transporter genes and barrier function, and small intestinal microbiota in broilers aiming to reveal the relationship among small intestinal microbiota, intestinal health and growth performance.

## MATERIALS AND METHODS

### Ethics statement

All experiment protocols were approved by the animal Care and Use Committee of Northwest A&F University (Protocol number: NWAFAC1008).

### Apidaecin Api-PR19 and antibiotics

The Apidaecin Api-PR19 was kindly provided by Aolinberer (Gansu, China) and is the subject of Chinese patents ZL2014-1-0654343.X. The details regarding Apidaecin Api-PR19 are listed in Table 1. Colistin sulfate and roxarsone (98% purity; Jiangsu Qiansheng Pharmaceutical Technology Co., Ltd, Jiangsu, China) was used as positive control in this study.

### Animals and experimental design

A total of 360 1-d-old healthy Arbor Acres broilers with no significant difference in body weight were provided by Dacheng company. In this study conducted with one-factor completely randomized design, 360 broilers were randomly assigned to 3 groups with 6 replicates and 20 broilers per replicate (half male and half female). All chickens were fed basal diet meeting nutritional requirements of broilers (Table 2) during starter and grower period. The antimicrobial peptides and antibiotics were added to the diet according to the manufacturer’s recommended concentration. The dietary treatments were as follows: control group (CON, basal diet), antibiotics group (AGP, control + 10 mg/kg colistin sulfate + 50 mg/kg roxarsone), and Apidaecin group (ABP, control + 330 mg/kg Apidaecin Api-PR19). All chickens were kept in an environmentally controlled poultry house with double-floor battery cages. The temperature in the poultry house was maintained at 35°C for the first week and gradually decreased to 27°C in the third week. The lighting program was set to 23-h photoperiod and broilers were allowed ad libitum access to fresh water and feeds. The experiment lasted for 35 d. On day 21 and 35, the broilers were weighted, and feed consumption was recorded by replicates. Average daily weight gain (ADG), average daily feed intake (ADFI), and feed conversion rate (FCR) were calculated.

### Sampling

After depriving of feed for a fasting period of 12 h. one bird with similar body size was selected from each replicate. Before sampling, chickens were injected with 3% sodium pentobarbital (25 mg/kg body weight; Sigma, St. Louis, MO, USA) and immediate dissection. Then 3 cm long segments were cut from the middle of duodenum, jejunum, and ileum, and fixed in 10% buffered formalin after removing chyme with saline solution. Next the duodenal, jejunal and ileal mixed contents and respective mucosa samples were collected and frozen with nitrogen immediately and stored at −80°C for further analysis.

### Determination of nutrient digestibility and digestive enzyme activity

We used an indicator approach to evaluate the nutrient digestibility of 35-d-old broilers. During day 28 to 35, broilers were fed basal diet with 0.5% Cr_2_O_3_ (Tianjin Zhiyuan Chemical Reagent Co., Ltd, Tianjin, China). After 5 days of dietary adaptation period, terminal ileal (segment of intestine from the ileocecal junction to the anterior ileum within a range of 15 cm) digesta of each broiler was collected in the next two days and stored in −20°C refrigerator immediately [10]. The digesta samples were dried in an oven at 65°C for 48 h to constant weight and ground for further assays. The samples of feed and digesta of each replicate were analyzed to determine digestibility of dry matter (DM; AOAC Method 930.15), crude protein (CP; AOAC Method 976.05) and crude fat (AOAC Method 960.39). Chromium was analyzed via UV absorption spectrophotometry. The following equation was used to calculate the ileal digestibility of DM, CP, and crude fat [11].


$$\text{Ileal digestibility }(\%)=[1-({\text{Cr}}_{2}{\text{O}}_{3}\;\text{in diet})/{\text{Cr}}_{2}{\text{O}}_{3}\;\text{in digesta})\times (\text{nutrient in digesta})/(\text{nutrient in diet})]\times 100$$

The activity of amylase, lipase, and trypsin in digesta of duodenum were measured with detection kits of NanJing JianCheng Bioengineering Institute.

### Determination of intestinal morphology

Duodenal, jejunal and ileal segments of broilers fixed in 10% buffered formalin were further dehydrated, cleared, and embedded in paraffin. Then paraffin sections were cut into 5 μm using a microtome, followed by staining hematoxylin and eosin. The villus height (VH) and crypt depth (CD) were measured in 8 randomly selected villi and crypt per slide using contrast microscope. Besides the ratio of VH to CD were calculated from each sample.

### Quantitative real-time RT-polymerase chain reaction (qRT-PCR) analyses

Total RNA of duodenal mucous membrane was extracted with TRIzol reagent (Hunan Accurate Biology Engineer Co., Ltd, Hunan, China) according to the manufacturer’s instructions. Spectrophotometer was used to determine RNA concentration and quality. Then total RNA was transcribed into cDNA using the Primescript RT master mix kit (Takara Bio Inc., Dalian, China). The polymerase chain reaction (PCR) primer sequences used in the study are shown in Table 3. qRT-PCR was conducted in an iCycler iQ5 multicolor real-time PCR detection system (Bio-Rad Laboratories, Shanghai, China) using SYBR Green PCR Master Mix (Takara, Dalian, China). Procedure was as followed: 95°C for 30 s, followed by 40 amplification cycles of 95°C for 15 s, 60°C for 30 s. Relative quantification of the target gene expression was quantified using the Livak method and normalized to the expression of Con group [12].

### Measurement of intestinal secretory immunoglobulin A by immunohistochemistry method

According to the previous study [13], immunohistochemistry method was applied to measure secretory immunoglobulin A (sIgA) content in intestine. Briefly, the intestine tissue was dewaxed, rehydrated, microwave irradiated, treated with 3% H_2_O_2_ at room temperature for 25 min, then the following steps were performed: rabbit serum blocking, primary antibody incubation overnight at 4°C (dilution ratio1:200), the secondary antibody incubation at room temperature for 50 min (dilution ratio 1:200), and staining by 3,3-diaminoben-zidine (DAB). The sIgA-positive areas were qualitatively analyzed by optical microscope and semi-quantitative analysis by Image-Pro Plus 6.0 software to calculate mean of optical density.

### 16S Ribosomal DNA gene sequencing

Microbial genomic DNA was extracted from the small intestinal contents using magnetic stool DNA kit (Nobleryder, Beijing, China) according to the manufacturer’s protocols. The V3+V4 hypervariable region of 16S rRNA gene was amplified with 515F (5′-GTGCCAGCMGCCGCGGTAA-3′) and 806R (5′-GGACTACHVGGGTWTCTAAT-3′) primer pairs [14]. DNA was separated by agarose gel electrophoresis and purified using a DNA Gel Extraction Kit (Qiagen, Valencia, CA, USA). TruSeq DNA PCR-Free Library Preparation Kit from Illumina company (San Diego, USA) was used to construct the library for sequencing. Operational taxonomic units (OTUs) were defined with a cut-off value of 97% and were then taxonomically classified by using Qiime software (Version 1.9.1) [15].

Alpha diversity including ACE, Chao1, Shannon and Simpson index were used to evaluate the richness and diversity of microbial community. Beta diversity was used to evaluate differences of samples in species composition, including principal coordinated analysis (PCoA) and distance matrices generated from unweighted Unifrac analysis. Besides, differential bacteria among the treatment groups were identified using linear discriminant analysis (LDA) effect size analysis (LDA>3, p<0.05). Redundancy analysis was performed to assess the correlation between the differential microbiota and the phenotypes of broilers.

### Statistical analysis

All data were performed by one-way analysis of variance using SPSS 25.0 software with replicates as experimental units and differences considered to be statistically significant at p<0.05 based on Duncan’s multiple comparison.

## RESULTS

### Growth performance

The effects of dietary ABP or AGP addition on ADFI, ADG. and FCR in broilers are shown in Table 4. AGP addition significantly increased (p<0.05) ADFI, ADG during in 1 to 35 d compared with CON group. Although ABP group had no significant difference in growth performance compared with CON group during 1 to 35 d, increased (p<0.05) ADFI, ADG and decreased (p<0.05) FCR were observed in ABP group during 1 to 21 d. In comparison with AGP group, ABP group had lower (p<0.05) ADFI, but there is no difference in ADG and FCR between ABP and AGP group during 1 to 35 d.

### Nutrient digestibility and digestive enzyme activity

Table 5 shows the nutrient digestibility of terminal ileum and duodenal digestive enzyme activities at 35 d. Both AGP and ABP treatment significantly improved (p<0.05) DM and CP digestibility of 35-d-old broilers compared with CON group. Dietary ABP significantly increased duodenal trypsin activity compared to the CON group (p<0.05), while had no effect on lipase and amylase (p>0.05).

### Intestinal morphology

**Figure 1:**
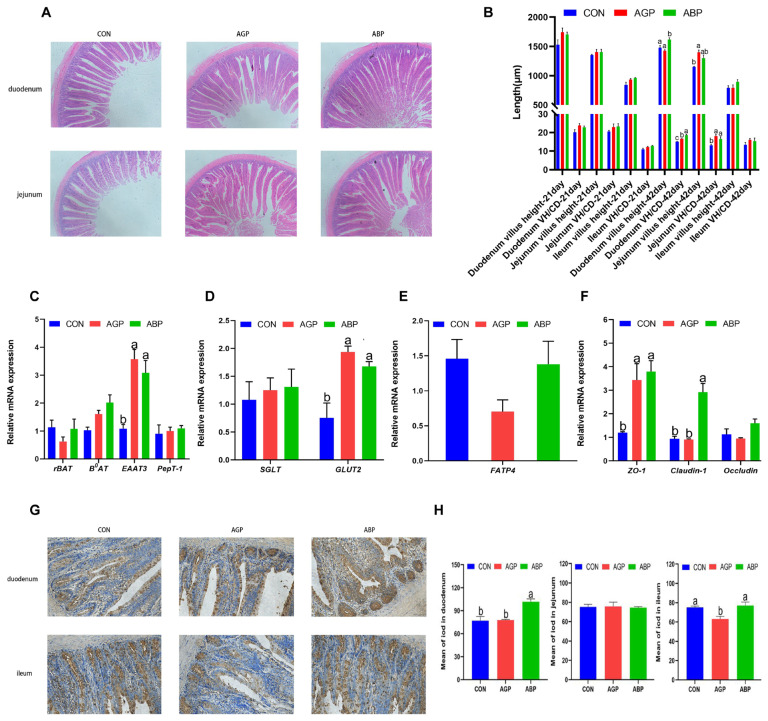
Effects of Api-PR19 and colistin sulfate on intestinal morphology, gene expression and secretory immunoglobulin A (sIgA) content. (A) Representative hematoxylin-eosin (HE) stain images and (B) villus height and villus height/crypt depth (VH/CD) in intestine of 21 and 35-d-old broilers. The relative mRNA expression of (C) amino acid transporters (*rBAT*, related to b^0,+^ amino acid transporter; *B^O^AT*, system B^0^ neutral AA transporter; EAAT-3, excitatory amino acid transporter-3), (D) glucose transporters (*SGLT*, Na^+^-dependent glucose transporters; *GLUT2*, glucose transporter-2), (E) fatty acid transporters (*FATP4*, fatty acid transporter protein-4), and (F) tight junction protein (*ZO-1*, zonula occludens-1; *Claudin-1*) in duodenum. (G) Representative immunohistochemical images and (H) sIgA content in intestine tissue of 35-d-old broilers. CON, control group; AGP, antibiotic growth promoter group (basal diet with 10 mg/kg colistin sulfate); ABP, antibacterial peptide group (basal diet with 330 mg/kg apidaecin); ^a–c^ Bars with different letters differ (p<0.05).

The effect of dietary AGP and ABP on small intestinal morphology of broilers at 21 and 35 d of age were evaluated. The results showed that there were no difference (p>0.05) on VH, CD, and the ratio of VH to CD (V/C) at 21 d of age among CON, AGP, and ABP groups. However, at 35 d of age, ABP group significantly increased (p<0.05) duodenal VH, decreased (p<0.05) CD and increased (p<0.01) the V/C compared to the CON and AGP group. In the jejunum, supplement of ABP significantly increased (p<0.05) V/C compared with CON group. There were no significant difference (p>0.05) in ileum VH, CD, and V/C among CON, AGP, and ABP groups (Figure 1A and 1B).

### Gene expression of duodenal mucosa

To investigate the effect of ABP on intestinal health related gene expression, we determined mRNA expression levels of duodenal nutrient transports and tight junction protein at 35 d, including amino acid transporter *rBAT*, *B^O^AT*, *EAAT3* and peptide transporter *Pept1*, glucose transporter *SGLT* and *GLUT2*, fatty acid transporter *FATP4*, tight junction protein *ZO-1*, *Claudin-1* and *Occludin*. Result shows that the expression level of *EAAT3* was significantly increased (p<0.05) in AGP and ABP group compared with CON group (Figure 1C). Supplement of ABP significantly improved (p<0.05) *GLUT2* expression level compared with CON group (Figure 1D). However, there were no significant differences (p>0.05) in *FATP4* expression level among CON, AGP, and ABP group (Figure 1E). Furthermore, both AGP and ABP supplementation significantly increased (p<0.05) *ZO-1* expression level compared with CON group. A higher (p<0.05) *claudin-1* expression level was found in ABP group when compared with CON and AGP group (Figure 1F).

### Intestinal sIgA content

We further measured sIgA content of duodenum, jejunum, and ileum. We found that diet supplemented with ABP significantly increased (p<0.05) duodenal sIgA content, there was no significant difference in jejunal sIgA content among three groups. While AGP treatment decreased (p<0.05) the ileal content of sIgA (Figure 1G, 1H).

### Microbial diversity and community in small intestinal

**Figure 2:**
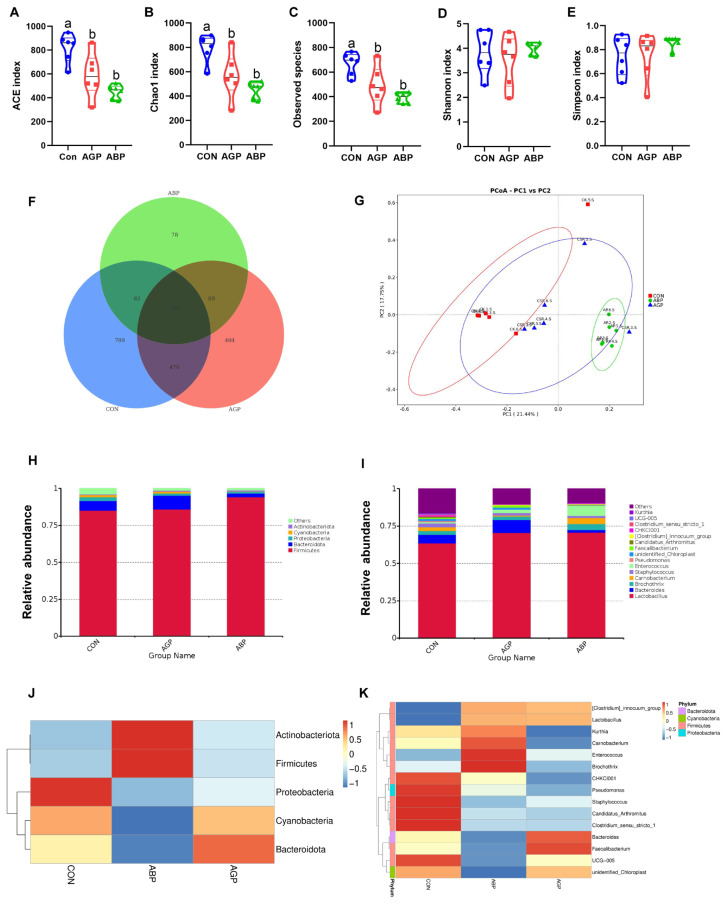
Effects of Api-PR19 and colistin sulfate on abundance of small intestine microbial diversity, structure, and abundance of small intestine microbial community. (A–E) Alpha diversity (ACE, Chao1, observed species, Shannon and Simpson index) of intestinal microbiota. (F) Venn diagram analyses based on the identified OTUs in CON, AGP, and ABP groups. (G) PCoA analyses identified differences in gut microbiota among CON, AGP, and ABP groups. Relative abundance and clustering heat map of (H and J) top 5 phyla and (I and K) top 15 genus among CON, AGP, and ABP groups. The abundance is expressed in terms of percentage of the total effective bacterial sequences in small intestinal samples. Relative abundance was Z score transformed. Warm and cold color indicates high and low abundance of microbiota respectively. CON, control group; AGP, antibiotic growth promoter group (basal diet with 10 mg/kg colistin sulfate); ABP, antibacterial peptide group (basal diet with 330 mg/kg apidaecin).

For determining different microbiological mechanisms of AGP and ABP on promoting growth performance, we then detected small intestinal microbial composition in broilers at 35 d. 16S rRNA gene sequencing results shows that both supplements of AGP and ABP significantly decreased Observed species, ACE and Chao1 indices compared with CON group (Figure 2A–2E). Moreover, the Venn diagram indicated that 799, 404, and 78 unique OTUs in the CON, AGP, and ABP groups, respectively (Figure 2F). The PCoA analysis based on unweighted unifrac at OTU level revealed that there was a clear separation in small intestinal microbial communities between CON and AGP groups (p = 0.023) or between CON and ABP groups (p = 0.003) or between ABP and AGP groups (p = 0.011). Besides, the ABP group clustered better than AGP group (Figure 2G). Taken together, these results indicated that supplement of ABP or AGP significantly altered microbial diversity and shaped a unique microbial community structure.

### Taxonomic composition of small intestinal microbiota

The phylum level taxonomic composition analysis showed that Firmicutes (88.36%) and Bacteroidota (6.13%) were the dominant bacteria in small intestinal microbiota of 35-d-old broilers, accounting for more than 90% of the total bacterial community (Figure 2H). At genus level, the top three genera in CON group were *Lactobacillus* (63.62%), *Bacteroides* (5.74%), and *Staphylococcus* (2.54%); those in AGP group were *Lactobacillus* (70.64%), *Bacteroides* (8.78%), and *Enterococcus* (2.25%); and those in ABP group were *Lactobacillus* (70.64%), *Enterococcus* (6.67%), and *Brochothrix* (4.00%) (Figure 2I). Heatmap and H-test analyses of the relative abundance of microbiota at phylum and genus levels among three groups showed that ABP group significantly enriched Firmicutes (p<0.05). At genus levels, dietary ABP also significantly increased the relative abundance of *Enterococcus* (p<0.05) compared with other two groups and decreased the relative abundance of *Clostridium_sensu_stricto_1* compared with CON group. Besides, AGP group had lower relative abundance of *Candidatus_Arthromitus* than CON group (Figure 2J, 2K; Table 6).

**Figure 3:**
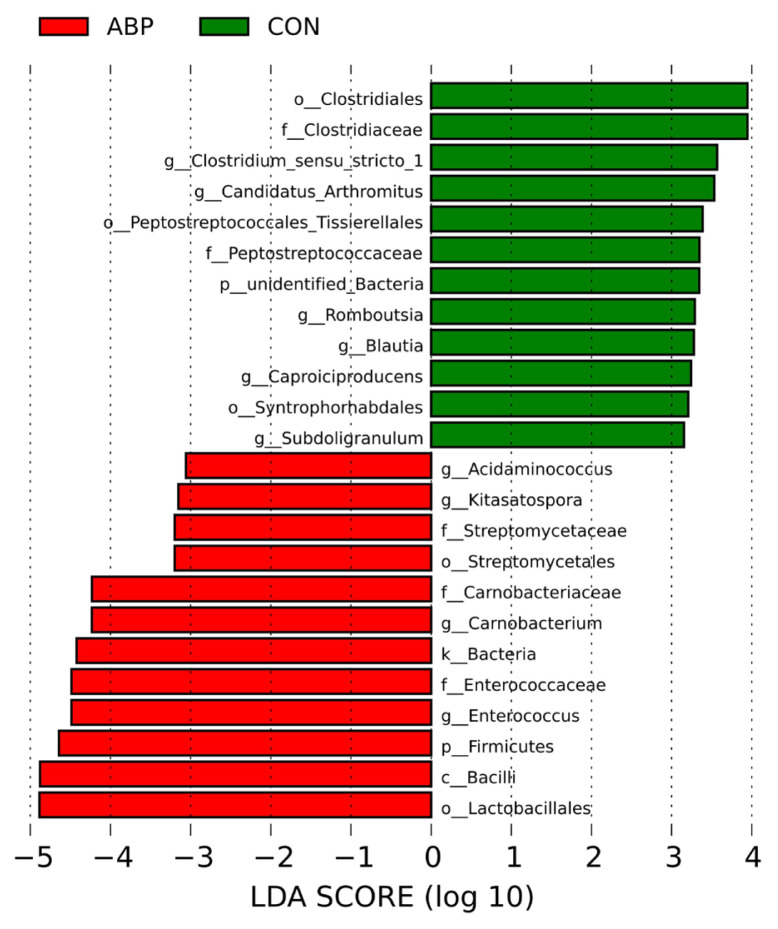
Differentially abundant intestinal microbial taxa identified by LEfSe analysis. Biomarker bacterial operational taxonomic units (OTUs) identified by Linear discriminant analysis effect size (LEfSe, LDA>3.0; p<0.05). Green bars represent enriched or exclusive OTUs in CON group, while red bars represent enriched or exclusive OTUs in ABP group. CON, control group; AGP, antibiotic growth promoter group (basal diet with 10 mg/kg colistin sulfate); ABP, antibacterial peptide group (basal diet with 330 mg/kg apidaecin).

In addition, differences in the composition of small intestinal microbiota were further analyzed by linear discriminant analysis effect size (LEfSe) method (p<0.05; LDA>3.0). *g_Clostridium_sensu_stricto_1*, *g_Candidatus_Arthromitus*, *g_Romboutsia*, *g_blautia*, *g_Caproiciproducens*, *g_Subdoligranulum* were enriched in CON group. *p_Firmicutes*, *g_Enterococcus*, *g_Carnobacterium*, *g_Kitasatospora*, *g_Acidaminococcus* were dominant in ABP group and there was no statistically different biomaker was found in the AGP group (Figure 3).

### Redundancy analysis

**Figure 4:**
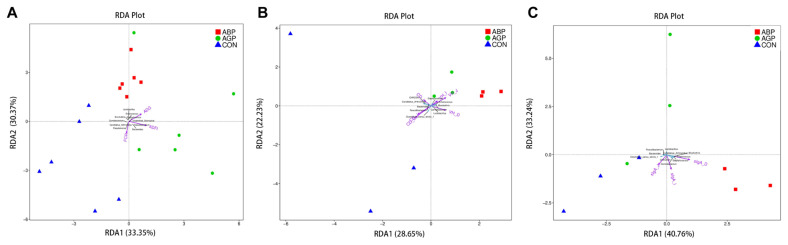
Redundancy analyses based on the identified differential genera and significantly altered phenotype. RDA analysis based on the identified differential species and (A) growth performance, (B) intestinal morphology and (C) secretory immunoglobulin A (sIgA) content of 35-d-old broiler. The angle between two lines was less than 90 degrees means positive correlation between differential species and identified altered phonetypes; the angle between two lines was more than 90 degrees means negative correlation between differential species and identified altered phonetypes. If the plot projection of one sample occurred in the positive direction of the extending line of the identified altered phenotypes, then the treatment of this sample could promote this phenotypic change. CON, control group; AGP, antibiotic growth promoter group (basal diet with 10 mg/kg colistin sulfate); ABP, antibacterial peptide group (basal diet with 330 mg/kg apidaecin).

We further performed redundancy analysis to identify the relationship among the differential genera of microbiota, treatment and performance. As a result, we found that ABP and AGP group were separately clustered in the area which was located at the positive direction of increased ADG and ADFI, as well as in the negative direction of increased FCR. While the contrary phenomenon was observed in CON group. These results showed that the intestinal microbiota variation mediated by ABP and AGP treatment was positively correlated with the growth performance (Figure 4A). Similar results were observed for analysis of intestinal morphology (Figure 4B). Moreover, the ABP group was clustered in the positive direction of increased sIgA content of duodenum and ileum and CON group were clustered in the positive direction of increased sIgA content of jejunum. However, AGP group show a negative direction of increased intestinal sIgA content. These results indicated that AGP supplementation was harmful to the content of intestinal sIgA while ABP treatment could increase the sIgA content (Figure 4C). Specifically, the ABP group was gathered near the positive direction of the extending line of the increased abundance of *Enterococcus*, which was positively related with ADG, ADFI, VH of duodenum, jejunum, and ileum and sIgA content of duodenum but negatively related with FCR and CD of duodenum, jejunum, and ileum. All results mentioned above indicated that increasing growth performance and intestinal health might be mediated by higher abundance of *Enterococcus* in ABP group.

## DISCUSSION

Antibacterial peptide as a novel feed additive had been proven to be a good alternative to AGP due to its natural antibacterial properties and low propensity to develop antibiotic resistance. Antimicrobial peptides are widely used in livestock and poultry production industry because of their beneficial effect of growth promoting, immune regulation and inhibition of pathogenic bacteria [16]. The present study is carried out to explore the effects of ABP Apidaecin Api-PR19 in poultry production.

In the present study, we found that supplementation of 330 mg/kg Apidaecin Api-PR19 can increase ADFI along with ADG and decrease FCR during early growth stage of Arbor Acres broilers, and the beneficial effect of Apidaecin Api-PR19 on growth performance was similar to AGP in the view of total growth phases. Similar to our finding, diet supplemented with different dose of pig ABPs could affect growth performance including ADFI, ADG, and FCR during starter period of Arbor Acres broilers [17]. In contrast to the results of our experiment, there was no effect of dietary supplementation of lactoferrin ABPs on growth performance of weaning pigs [18]. The variation of results might be because of the type of the ABPs, level of added dose and species of the experimental animal.

Like many other studies, AGP along with Apidaecin Api-PR19 improved growth performance of broilers, as reflected by greater DM and CP digestibility in the present study. Like our results, 0.75% potato ABP significantly increased retention of DM and CP of Ross broilers [19]. Improved digestibility of nutrients was also observed in weaned piglets fed diet with colistin sulfate [20]. The small intestine is the main place where nutrients are digested and absorbed. It is rich in digestive enzymes that break down indigestible macromolecular nutrients into small molecules which are easier used by hosts. Deficiency of endogenous enzymes results in a decrease in nutrient digestibility. Various feed additives have been proved to have positive effects on small intestinal digestive enzyme activity [21]. In the present study, dietary supplementation of ABP Api-PR19 significantly increased duodenal trypsin activity. Previous study has shown a positive result that dietary recombinant plectasin significantly increased duodenal trypsin activity of 42-d-old Arbor Acres broilers [22]. Improvement of intestinal digestive enzymes activity may be due to the antimicrobial effect of ABP [23], so further measurement of more digestive enzyme in the whole small intestine with a larger population is necessary.

The integrity of the intestinal physical barrier is fundamental to the absorption of nutrients and usually can be assessed by indexes such as intestinal morphology and tight junction proteins. The VH, CD, VH CD ratio are the key indicators which define the small intestinal function. Specifically, the higher the VH, the greater the area in contact with nutrients. Crypts as villus factories protect intestinal stem cells and enhance villus renewal [24]. In the current study, we found that dietary ABP Api-PR19 significantly improved duodenal, jejunal morphology with higher VH and decreased CD as well as their ratio. In line with the present study, diets supplemented with pig ABPs increased VH of duodenum and jejunum of Arbor Acre broilers. Oral administration of rabbit sacculus rotundus antimicrobial peptides increased VH of duodenum and jejunum of leghorn chicken [25]. But research on the effect of orally administrated different dosages of ABP J25 on mice showed that adding 18.2 mg/kg recombinant MccJ25 had no significant effect on VH and CD, while V/C was significantly decreased [26]. It is possible that the addition of high dose ABP may increase the risk of toxicity which damage intestinal morphology and function. Therefore, the biosecurity of ABPs, especially the cytotoxic effect is a crucial indicator to design and develop novel ABPs [27].

The tight junctions in the intestinal epithelium are important components of dynamic barrier structure, which blocks many microbes and antigenic substances from the intestinal lumen into the body by regulating the permeability between cells. Zonula occludens (*ZO-1*) maintains epithelial barrier function, epithelial polarity and participates in material transport. Claudin protein is a specific protein to ensure tight junction permeability. In the present study, we found that diet supplemented with Api-PR19 significantly increased gene expression of *ZO-1* and *Claudin-1* in the duodenum of broilers. Previous studies attributed that ABPs cLFchimera significantly upregulated the expression of *Claudin-1* in the intestine of broilers challenged by necrotic enteritis [28]. Similarly, diet supplement with pratt and full-tide ABPs significantly upregulated the expression of *ZO-1* [29]. The improvement of intestinal physical barrier may be due to inhibition of pathogen colonization by antimicrobial peptides, which reduce inflammatory response of epithelial cells [30]

Small molecule nutrients in the intestine are transported into cells by corresponding nutrient transporters. The expression of nutrient transporters is regulated by the concentration of nutrient substrates in the intestinal lumen. In the present study, we found that ABP Api-PR19 treatment significantly upregulated expression of *EAAT3*, *GLUT2* in the duodenum. Excitatory amino acid transporter 3 (*EAAT3*) is a transporter responsible for acidic amino acid including glutamate, which is a major oxidative fuel for the intestine. Glucose transporter 2 (*GLUT2*) together with *SGLT1* oversees the absorption and transport of glucose in the intestine. Glucose binds to *GLUT2*, a specific carrier on the microvilli of the small intestine and is transported to the blood via the way of easy diffusion [31]. According to the previous study, dietary ABP Api-PR19 significantly upregulated expression of *GLUT2* in duodenum of 21-d-old broilers [9]. Upregulation of gene expression of nutrient transporters is a mechanism to utilize abundant resources which could be partly explained by intestinal adaptation to changes in nutrient substrate concentrations.

According to the results of previous studies, antibiotics could inhibit intestinal pathogens, and reduce energy loss induced by inflammation to maintain balance of intestinal microflora and enhanced microbial synthesis pathways of nutrients and secondary metabolites [32]. As reflected in results of the present study, the antibiotic growth performance indeed enhances intestinal digestion, absorption, and utilization of nutrients. But potential danger followed because ileal sIgA content was significantly decreased in AGP group. sIgA is the most abundant immunoglobulin in the body and plays an important role in intestinal mucosal immunity, including immune rejection, antigen presentation and interaction with intestinal microbiota. Lack of sIgA would lead to deterioration of mucosal immunity and bacterial overgrowth, adherence, and translocation. The decrease in sIgA in the AGP group may be due to the excessive inhibition of gut bacteria, including probiotics [33]. In contrast to the mechanism of antibiotics, ABP Api-PR19 significantly increased sIgA content in the duodenum. Similarly, higher content of sIgA in duodenum was found in broilers when given diets or drinking supplemented with pig ABP [17]. The previous study also reported that ABPs played a crucial role not only in controlling the microbiome but also in gut innate and adaptive immunity [34].

We further detected the influence of ABP Api-PR19 and antibiotic on small intestinal microbiota to reveal their microbial mechanisms for growth promotion. Even though small intestinal microbiota has fewer members and lower diversity than that in the hindgut, they develop important functions for nutrients digestion and absorption [35]. However, few studies have considered the roles of small intestinal microbiota of broilers chickens. Hence, in consideration of microbial similarity in diversity and function among duodenum, jejunum, and ileum [32], we regarded small intestine as a whole to study the effects of Api-PR19 on gut microbiota.

In the present study, ABP and AGP treatment significantly decreased Shannon and Simpson index of intestinal microbiota, and beta-diversity analysis also showed significant clustering among treatment groups, indicating that ABP and AGP treatment significantly altered small intestinal microbiota community. Further analysis of microbiota taxonomical composition showed that Firmicutes, Bacteroidota and Proteobacteria are the major phyla of small intestinal microbiota in broilers which was consistent with previous studies [32]. Diet supplement with ABP Api-PR19 significantly increased relative abundance of Firmicutes of broilers compared with CON and AGP group. Firmicutes is important symbiotic bacteria in human and animal intestines which could utilize dietary fiber and produce the corresponding metabolites.

At genus levels, *Enterococcus*, *Carnobacterium*, *Kitasatospora* and *Acidaminococcus* were enriched in broilers fed with ABP Api-PR19. *Enterococcus* including *enterococcus faecium* and *enterococcus faecalis* are generally considered as beneficial microbes since they can maintain intestinal health and promote immune function, thereby enhancing animal growth [36]. *Carnobacterium* is a commensal bacteria in fish gastrointestinal tract, some strains among which can produce bacteriocins to against pathogenic bacteria [37]. *Kitasatospora* is the antibiotic-producing genera from *Streptomycetaceae* and produces a variety of natural antibiotics [38]. Previous study had shown that there was strongly correlation between apparent metabolizable energy of broilers and *Acidaminococcus* sp. [39]. *Candidatus Arthromitus* was reported to regulate T helper (Th17) cells differentiation IgA plasma cells induction and intestinal IgA secretions [40]. It was inhibited in AGP group in our study, which lead to a decrease in intestinal sIgA content. Unlike the AGP group which indiscriminately killed bacteria including probiotics and pathogenic bacteria, ABP treatment altered intestinal bacteria in a gentler way and might, reduce pathogenic bacteria while increasing the relative abundance of probiotic bacteria. Meanwhile, we found that relative abundance of *Clostridium_sensu_stricto_1* which was usually increased in necrotic enteritis was significantly decreased in ABP and AGP group compared with CON group. These results indicated that ABP could reduce the risk of necrotizing enteritis infection in our study.

Intestinal microbiota alteration may affect host growth performance, intestinal development, and immunity. Hence, we performed redundancy analysis to explore the relationship between differential genera and growth performance, intestinal development, or immune function. The results showed that several dominant genera in ABP group, especially *enterococcus* was significantly positively correlated with growth performance, intestinal VH and content of sIgA. In a word, Apidaecin Api-PR19 synergizes with the small intestinal microbiota community to improve growth performance, maintain intestinal health and promote intestinal development.

At last, the current study illustrated application effects of Apidaecin Api-PR19 in broilers. These findings implied that intestinal health and growth performance was promoted by antimicrobial peptides Api-PR19. Compared with AGP, Api-PR19 was a more green and efficient feed additive and might be a qualified candidate alternative of AGP. However, further trials need to be carried out to validate the function of single strains or evaluate antibiotic resistance genes in gut and faeces in the future.

## CONCLUSION

In summary, dietary 330 mg/kg Apidaecin Api-PR19 addition inhibited the colonization of intestinal pathogens and increased the abundance of beneficial bacteria especially enterococcus, which improved growth performance, intestinal development, absorption, and immune function.

## Figures and Tables

**Table 1:** Physical and chemical parameters of Api-PR19

Item	Api-PR19
Amino acid composition	PRVRRPVYIPQPRPPHPRL
Isoelectric point	12.18
Charge number (+)	5
Total hydrophilic index	−1.316
Coefficient of fat	71.58
Beaumont index (kcal/mol)	3.52
Antibacterial activity score	0.802

**Table 2:** Ingredients and chemical composition of the basal diet (as-fed basis %)

Item	1 to 21 d	22 to 35 d
Ingredients (%)		
Corn	55.42	54.44
Soybean meal	27.64	22.69
Flour	6.00	8.00
Cottonseed meal	3.00	3.00
Corn gluten meal	2.00	4.00
DL-Methionine	0.18	0.11
Threonine	0.10	0.06
Lysine-HCL (70%)	0.54	0.51
Soybean oil	0.84	3.51
Limestone	1.39	1.58
Dicalcium phosphate	1.56	0.96
Choline chloride (60%)	0.10	0.10
Sodium chloride	0.30	0.30
Vitamin-mineral premix^1)^	0.93	0.74
Total	100	100
Nutrient level^2)^		
ME (MJ/kg)	12.01	12.84
CP (%)	20.04	18.12
Ca (%)	1.00	0.90
Total P (%)	0.67	0.63
Available P (%)	0.45	0.37
Lys (%)	1.15	1.00
Met (%)	0.50	0.40

ME, metabolizable energy; CP, crude protein.

1) Provided per kilogram of diet: vitamin A, 12,000 IU; vitamin B_1_, 5 mg; vitamin B_2_, 9.6 mg; vitamin B_12_, 0.02 mg; vitamin D_3_, 3100 IU; vitamin E, 20 IU; vitamin K_3_, 4 mg; biotin, 0.2 mg; niacin, 60 mg; folic acid, 1 mg; Cu, 8 mg; Fe, 70 mg; Mn, 110mg; Zn, 110 mg; Se, 0.3 mg.

2) The nutrient levels were calculated values.

**Table 3:** Primer sequences used for quantitative reverse transcription polymerase chain reaction

Target gene	Nucleotide sequence (5′–3′)	Accession no.
*β-actin*	F: AACACCCACACCCCTGTGAT	NM_205518.2
	R: TGAGTCAAGCGCCAAAAGAA	
*EAAT3*	F: ACCCTTTTGCCTTGGAAACT	XM_046936555.1
	R: TTGAGATGTTTGCGTGAAG	
*BOAT*	F: TATCCTGGCTGGGTCTATGC	XM_040663289.2
	R: AGGCCTGTACGATCCCTTCT	
*rBAT*	F: CCCAACAACTGGGTGAGTGT	XM_040667709
	R: AAGTCCGGCTGTTCTTTCCC	
*PepT-1*	F: CGCGTCACTTGCCTTTTTGA	NM_076686.7
	R: TTTGACGGCACATGGCAAAG	
*GLUT2*	F: TCCTGCTAAGTGATCCGATGC	NM_207178.2
	R: CCCTTCCAACCCAAACCA	
*SGLT1*	F: GCTTCACTCTTTGCCAGTAACA	XM_046928028.1
	R: CATTCCATTCATACCCTCCAAT	
*FATP4*	F: ATACCTCTGGCACTACGGGAAT	XM_046929199.1
	R: CATACATCACATCATCGGGTCT	
*ZO-1*	F: CTTCAGGTGTTTCTCTTCCTCCTC	XM_046925214.1
	R: CTGTGGTTTCATGGCTGGATC	
*Claudin-1*	F: GCAGATCCAGTGCAAGGTGTA	NM_001013611.2
	R: CACTTCATGCCCGTCACAG	
*Occludin*	F: AGGTGAAGGCGTGTTTCCAT	XM_012873661.3
	R: CATCCTCGCATCATCCGTGA	

**Table 4:** Effects of dietary Api-PR19 or colistin sulfate addition on growth performance in broilers

Items	Treatment^1)^			SEM	p-value

	CON	AGP	ABP		
Day 1 to 21					
ADFI (g)	40.57^c^	45.67^a^	42.92^b^	0.74	<0.001
ADG (g)	28.90^c^	34.12^a^	30.60^b^	0.73	<0.001
FCR^2)^	1.40^a^	1.34^b^	1.38^b^	0.01	0.010
Day 21 to 35					
ADFI (g)	113.00^b^	120.20^a^	110.43^b^	2.31	0.020
ADG (g)	73.40	77.12	73.90	3.96	0.610
FCR	1.54	1.52	1.51	0.09	0.920
Day 1 to 35					
ADFI (g)	66.37^b^	71.20^a^	66.81^b^	1.28	0.030
ADG (g)	44.78^b^	49.61^a^	46.11^ab^	1.66	0.030
FCR	1.49	1.44	1.46	0.05	0.660

The data are the means of 6 replicates per treatment.

AGP, antibiotic growth promoter (colistin sulfate); ABP, antibacterial peptide (Api-PR19); SEM, standard error of mean; ADFI, average daily feed intake; ADG, average daily gain; FCR, feed conversion ratio.

1) CON, basal diet; AGP, basal diet with 10 mg/kg colistin sulfate; ABP, basal diet with 330 mg/kg apidaecin.

2) FCR = average daily feed intake (g) / average daily gain (g).

a–c Value in a row with no common superscripts differ significantly (p<0.05).

**Table 5:** Effects of dietary Api-PR19 or colistin sulfate on nutrient digestibility and enzyme activity in duodenum of 35-d-old broilers

Items	Treatment^1)^			SEM	p-value

	CON	AGP	ABP		
Nutrient digestibility (%)					
Dry matter	67.67^b^	70.58^a^	69.74^a^	0.82	0.031
Crude protein	80.52^b^	85.06^a^	84.44^a^	1.47	0.015
Crude fat	71.11	71.92	73.59	1.83	0.434
Enzyme activity (U/mg)					
Trypsin	2,569.24^b^	3,042.27^ab^	3,372.47^a^	245.58	0.021
Lipase	13.09	10.05	12.23	3.34	0.651
Amylase	3.29	3.93	4.37	1.15	0.649

The data are the means of 6 replicates per treatment.

AGP, antibiotic growth promoter (colistin sulfate); ABP, antibacterial peptide (Api-PR19); SEM, standard error of mean.

1) CON, basal diet; AGP, basal diet with 10 mg/kg colistin sulfate; ABP, basal diet with 330 mg/kg apidaecin.

a,b Value in a row with no common superscripts differ significantly (p<0.05).

**Table 6:** Relative abundance of intestinal microbiota at phylum and genus levels

Items	Treatment^1)^			SEM	p-value

	CON	AGP	ABP		
Phylum					
Firmicutes	85.03^b^	85.87^b^	94.20^a^	3.06	0.005
Bacteroidota	6.64	9.32	2.44	3.42	0.161
Proteobacteria	2.53	1.43	1.11	1.49	0.834
Cyanobacteria	1.36	1.28	0.18	0.73	0.229
Actinobacteriota	0.57	0.61	0.85	0.31	0.642
Genus					
*Lactobacillus*	63.61	70.49	70.65	7.80	0.599
*Bacteroides*	5.74	8.78	1.86	3.26	0.139
*Brochothrix*	2.54	2.15	4.00	2.55	0.752
*Carnobacterium*	2.44	0.74	3.94	2.36	0.418
*Staphylococcus*	2.54	1.75	1.55	2.04	0.877
*Enterococcus*	0.89^b^	2.25^b^	6.67^a^	1.59	0.006
*Pseudomonas*	0.80	0.09	0.31	0.67	0.096
*unidentified_Chloroplast*	1.34	1.27	0.17	0.74	0.237
*Faecalibacterium*	0.72	1.29	0.21	0.61	0.241
*Candidatus_Arthromitus*	0.80^a^	0.05^b^	0.10^ab^	0.26	0.005
*[Clostridium]_innocuum_group*	0.11	0.49	0.50	0.34	0.439
*CHKCI001*	0.58	0.11	0.34	0.24	0.143
*Clostridium_sensu_stricto_1*	0.72^a^	0.01^b^	0.01^b^	0.22	0.003
*UCG-005*	0.53	0.28	0.07	0.20	0.119
*Kurthia*	0.25	0.03	0.33	0.27	0.538

The data are the means of 6 replicates per treatment.

AGP, antibiotic growth promoter (colistin sulfate); ABP, antibacterial peptide (Api-PR19); SEM, standard error of mean.

1) CON, basal diet; AGP, basal diet with 10 mg/kg colistin sulfate; ABP, basal diet with 330 mg/kg apidaecin.

a,b Value in a row with no common superscripts differ significantly (p<0.05).
